# Co-Delivery of 8-Hydroxyquinoline Glycoconjugates and Doxorubicin by Supramolecular Hydrogel Based on α-Cyclodextrin and pH-Responsive Micelles for Enhanced Tumor Treatment

**DOI:** 10.3390/pharmaceutics14112490

**Published:** 2022-11-17

**Authors:** Adrian Domiński, Tomasz Konieczny, Marcin Godzierz, Marta Musioł, Henryk Janeczek, Aleksander Foryś, Monika Domińska, Gabriela Pastuch-Gawołek, Tomasz Piotrowski, Piotr Kurcok

**Affiliations:** 1 Centre of Polymer and Carbon Materials, Polish Academy of Sciences, 34, M. Curie-Skłodowskiej St, 41-819 Zabrze, Poland; 2 Department of Organic Chemistry, Bioorganic Chemistry and Biotechnology, Faculty of Chemistry, Silesian University of Technology, Krzywoustego 4, 44-100 Gliwice, Poland; 3 Biotechnology Centre, Silesian University of Technology, B. Krzywoustego 8, 44-100 Gliwice, Poland; 4 Department of Chemical Organic Technology and Petrochemistry, Faculty of Chemistry, Silesian University of Technology, Bolesława Krzywoustego 4, 44-100 Gliwice, Poland

**Keywords:** pH-responsive, drug release, supramolecular hydrogel, α-cyclodextrin, quinoline glycoconjugates, Warburg effect

## Abstract

The sustained release of multiple anti-cancer drugs using a single delivery carrier to achieve a synergistic antitumor effect remains challenging in biomaterials and pharmaceutics science. In this study, a supramolecular hydrogel based on the host–guest complexes between pH-responsive micelle derived poly(ethylene glycol) chains and α-cyclodextrin was designed for codelivery of two kinds of anti-cancer agents, hydrophilic 8-hydroxyquinoline glycoconjugate and hydrophobic doxorubicin. The host–guest interactions were characterized using X-ray diffraction and differential scanning calorimetry techniques. The resultant supramolecular hydrogel showed thixotropic properties, which are advantageous to drug delivery systems. In vitro release studies revealed that the supramolecular hydrogel exhibited faster drug release profiles in acidic conditions. The MTT assay demonstrated a synergistic cancer cell proliferation inhibition of DOX/8HQ-Glu mixture. In vitro cytotoxicity studies indicated excellent biocompatibility of the supramolecular hydrogel matrix, whereas the DOX/8HQ-Glu-loaded supramolecular hydrogel showed a sustained inhibition efficacy against cancer cells. The codelivery of hydrophobic anti-cancer drugs and hydrophilic anti-cancer drug glycoconjugates via a pH-responsive supramolecular hydrogel opens up new possibilities for the development of an effective cancer treatment based on the tumor-specific Warburg effect.

## 1. Introduction

Supramolecular hydrogels (SMHs) based on host–guest interactions are considered an extremely interesting group of soft materials with potential applications in the biomedical field [1,2]. In contrast to conventional polymeric hydrogels crosslinked via covalent linkages, the supramolecular systems are a result of dynamic physical interactions, such as van der Waals, host–guest and π-π dipolar interactions, hydrogen bonding, or hydrophobic effects [3]. These dynamic physical interactions impart SMHs with self-healing properties, stimuli-responsive behaviors, and adjustable mechanical properties, since the noncovalent interactions are dynamic and reversible [4]. Moreover, SMHs are thixotropic, which make them highly promising injectable implants as drug delivery carriers, because the supramolecular hydrogel might be injected into a specific place under shear forces, and turned to gel in situ after withdrawing the shear forces [5,6]. SMHs based on the poly(pseudo)rotaxane formation between α-cyclodextrin (α-CD) and poly(ethylene glycol) (PEG) have gained considerable attention as an attractive platform for engineering novel functional materials for drug delivery applications [7,8]. The cyclodextrins are toroid shape macrocyclic oligosaccharides, consisting of 6, 7, or 8 D(+)-glucose units linked via alfa-1-4-glucosidic bonds, called α-, β-, or γ-cyclodextrin, respectively [9]. The host–guest interactions allow the PEG chains to penetrate the cavity of α-CD to form poly(pseudo)rotaxanes, which are enhanced by hydrogen bonds between neighboring α-CD molecules threaded on the same PEG chain. The supramolecular hydrogel is physically crosslinked via the channel-type crystalline aggregation of α-CD/PEG inclusion complexes [10]. Nanoparticles based on PEGylated amphiphilic diblock copolymers have been successfully used as a building platform to create SMHs with α-CD [7,11]. Moreover, the multi-functionality of polymeric nanoparticles could be easily adaptable to many drug delivery systems. By the introduction of stimuli-responsive groups into nanoparticle building segments, the supramolecular hydrogel can be designed to take advantage of various pathophysiological markers characteristic of tumor tissues and release the proper amount of anti-cancer agents in response to signals caused by neoplastic cells [12].

One of the hallmarks of cancer cells is that they take up sugar derivates such as glucose, galactose, glucosamine, or mannose at higher rates than healthy cells, yet less sugars are involved in the oxidative phosphorylation process [13]. The deficient oxidation of sugar derivatives through the glycolysis pathway leads to the accumulated pyruvate being mainly transformed in lactic acid fermentation, resulting in acidification of the tumor microenvironment [14]. However, this incomplete oxidation of sugars is preferred by cancer cells and is effectively employed for ATP production. As a consequence, tumor tissues demand increased glucose or other sugar derivatives uptake to maintain their fast growth rate and increase proliferation [15]. The enhanced glucose and other monosaccharides uptake by neoplastic cells is related to the overexpression of glucose transporters [16]. This atypical relationship between the increased proliferation of neoplastic cells and the high rate of monosaccharide uptake, with simultaneous acidification of the tumor tissues microenvironment, caused by lactate secretion, is known as the Warburg effect and has been found in almost all kinds of cancers [17]. Considering the tumor-specific Warburg effect, researchers have exploited it in drug delivery systems to achieve tumor-oriented nanocarriers or improve the efficacy and selectivity of cancer therapy e.g., developing ligand-decorated [18] or pH-sensitive nanocarriers [19], or by adding a sugar moiety into the anti-cancer agent structure (glycoconjugation) [20].

Injectable hydrogels can successfully load hydrophilic drugs by mixing the anti-cancer agents with the aqueous SMH precursor solution, and the anti-cancer drug loaded SMH can be easily administrated to the patient in a non-invasive manner. However, most anti-cancer drugs are hydrophobic, and these are not capable to be loaded into a hydrophilic hydrogel matrix. To overcome these challenges, the supramolecular hydrogel based on α-CD/PEGylated nanocarriers has been developed, which might improve conventional chemotherapy. Thus, such supramolecular hydrogels provide an opportunity for simultaneous loading and delivery of hydrophilic and hydrophobic anti-cancer agents to enhance cancer therapy. Therefore, pH-responsive SMHs, designed to degrade in the acidic tumor environment, should be able to release and selectively deliver their therapeutic cargo, in a controlled manner, into the tumor and surrounding tissues. Yao et al. [21] prepared pH-responsive supramolecular hydrogel from glycol chitosan-Pluronic F127 conjugate (GC-PF127)-based micelles and α-CD for local tumor chemotherapy. The supramolecular hydrogels release drug-loaded micelles in response to tumor acidic microenvironment via the dissociation of the hydrogel matrix. Moreover, the treatment of H22 tumor-bearing mice revealed increased doxorubicin (DOX) accumulation in tumor tissues and simultaneously limited drug accumulation in healthy tissues. In other studies, Ni and coworkers [22] reported supramolecular hydrogel based on a three-armed star copolymer, with arms consisting of PEG and poly(ε-caprolactone) blocks linked via acid-cleavable acetal bonds and α-cyclodextrin for pH-triggered drug delivery. Supramolecular hydrogel built from PEGylated doxorubicin and α-CD was also reported as an efficient pH-responsive system in cancer treatment [23]. Furthermore, SMH have also been demonstrated as efficient systems for the codelivery of two various anti-cancer drugs for sustained synergistic tumor therapy [24,25,26,27].

Very recently, our group reported that a combination of pH-sensitive nanocarriers encapsulated with glucose or galactose 8-hydroxyquinoline (8HQ) conjugates notably improve selectivity to inhibit the proliferation of neoplastic cells, since both vectors take advantage of the tumor-specific Warburg effect [28,29]. The 8-hydroxyquinoline (8-HQ) is an important organic structural motif found in numerous bioactive natural compounds and synthetic pharmacological agents [30]. The 8-HQ scaffold was successfully applied to design anti-cancer therapeutics used in the treatment of the broad spectrum of solid tumors. 8-HQ is an ionophore whose anti-cancer activity is attributed to complexing metal ions for which neoplastic tissues have an increased demand [31]. However, the free 8-HQ has poor solubility, pharmacokinetic parameters, and bioavailability. Therefore, the 8-HQ derivatives have been modified by adding a sugar unit to improve pharmacokinetic parameters and increase selectivity, by exploiting the overexpression of GLUT transporters in the tumor tissues [32,33]. Reports on combination therapy consisting of 8-hydroxyquinoline and conventional anti-cancer drugs showed great therapeutic outcomes for breast cancer treatment in mouse models [34,35,36].

**Scheme 1:**
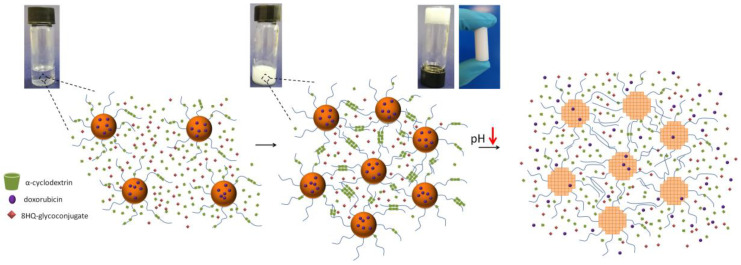
Schematic formation, drug loading, and environment triggered drugs release of pH-responsive supramolecular hydrogel.

Therefore, in the present work, we focused on developing supramolecular hydrogels for the pH-triggered codelivery of 8-hydroxyquinoline glycoconjugates and doxorubicin. The pH-sensitive biodegradable amphiphilic poly(ethylene glycol)-b-polycarbonate was synthesized via ring-opening polymerization of six-membered cyclic carbonate functionalized with a ketal moiety. The amphiphilic copolymer self-organizes into nanoscale micelles able to encapsulate DOX. Afterward, the solution of α-CD and 8HQ-glycoconjugates was mixed with an as-obtained, drug-loaded micelle dispersion, forcing host–guest complexation by threading α-CD onto the PEG chains localized in the outer micelle shell, and forming supramolecular hydrogels via physically crosslinked channel-type crystalline aggregation of the inclusion complexes (Scheme 1). Thereby, both DOX and glycoconjugates could be loaded in situ, forming a supramolecular hydrogel matrix that could be evaluated as a “smart” drug delivery device. The cytotoxicity of a free supramolecular hydrogel and drugs-loaded system were studied against both cancer (MCF-7) and normal cells (NHDF-Neo) by using the MTT assay.

## 2. Materials and Methods

### 2.1. Materials

Dichloromethane and toluene were purchased from Sigma-Aldrich (Steinheim, Germany) and dried using an Inert Puresolv Micro apparatus equipped with alumina columns. Tetrahydrofuran (THF) (Sigma-Aldrich, (Steinheim, Germany)) was distilled over a potassium-sodium alloy. Diethyl ether (VWR Chemicals, Fontenary-Sous-Bois, France) was used as received. Pentaerythritol, *p*-toluenesulfonic acid monohydrate and 2,2-dimethoxypropane were purchased from Sigma-Aldrich (Steinheim, Germany) and used as received. DOWEX-MARATHON and Dowex 50WX8, both from Sigma-Aldrich (Steinheim, Germany), were washed with THF before use. 1,8-Diazabicyclo[5.4.0]undec-7-ene (DBU, Sigma-Aldrich, Steinheim, Germany) was dried over CaH_2_, vacuum distilled, and stored in the glovebox. Poly(ethylene glycol) monomethyl ether (mPEG, Mn = 5000 g mol^−1^, Sigma-Aldrich, Steinheim, Germany) was dried via azeotropic distillation from dry toluene. Trimethylene carbonate (TMC) (HUIZHOU Foryou Medical Devices Co., Ltd., China) was recrystallized from ethyl acetate, dried under vacuum at room temperature (rt), and stored in a glovebox. α-CD (TCI Chemicals, Belgium) was dried overnight at room temperature in a vacuum oven before use. *N*-3,5-bis(trifluoromethyl)phenyl-*N*’-cyclohexylamine thiourea (TU) was prepared, as described in the literature [37]. Doxorubicin hydrochloride was purchased from LC Laboratories and deprotonated according to literature protocol [38]. The detailed preparation procedure of six-membered cyclic carbonate, functionalized with a ketal moiety (KPC, 9,9-dimethyl-2,4,8,10-tetraoxaspiro[5.5]undecan-3-one), was reported previously [29]. Model glycoconjugate (Figure S1) for in vitro cytotoxicity studies, i.e., 6-(4-(8-quinolinyloxymethyl)-1H-1,2,3-triazol-1-yl)-6-deoxy-D-glucopyranose (8HQ-Glu) was synthesized as described earlier [39].

#### Cell Lines

The human breast adenocarcinoma cell line (MCF-7) was received from collections at the Maria Skłodowska-Curie Memorial Cancer Center and Institute of Oncology (Gliwice, Poland). The Normal Human Dermal Fibroblasts—Neonatal (NHDF-Neo) were purchased from LONZA (Cat. No. CC-2509, Lonza, Poland). The culture media consisted of DMEM + F12 medium, with 5% antibiotics (penicillin and streptomycin) and 10% fetal bovine serum. The culture media was bought from HyClone. Fetal bovine serum (FBS) was purchased from Eurx, Poland. Antibiotic Antimycotic Solution (100×) was purchased from Sigma-Aldrich, Germany.

### 2.2. Synthesis of mPEG-b-PKPC and mPEG-b-PTMC

The polymerizations were carried out under an inert nitrogen atmosphere in a glovebox. The amphiphilic diblock copolymer was synthesized via polymerization of 9,9-dimethyl-2,4,8,10-tetraoxaspiro[5.5]undecan-3-one, using mPEG_5k_ as an initiator and DBU/TU as a catalytic system at room temperature. Briefly, KPC (2 g, 9.88 mmol, 20 equiv.) and TU (0.37 g, 0.98 mmol, 2 equiv.) were charged into the vial and dissolved in 15 mL of dry CH_2_Cl_2_. After solubilization, the solution containing mPEG (2.47 g, 0.49 mmol, 1 equiv.) and DBU (0.02 g, 0.09 mmol, 0.2 equiv.) was added to 10 mL CH_2_Cl_2_. The conversion of the monomer was determined using ^1^H NMR spectroscopy. After the reaction was completed, the polymerization was quenched by acidification with Dowex 50WX8 ion-exchange resin and the copolymer was precipitated in diethyl ether. The precipitated copolymer was dried, under a vacuum, to constant weight. The resulting mPEG-b-PKPC was stored in the glovebox. ^1^H NMR (CDCl_3_, 600 MHz): δ 1.42 ppm (6nH, CH_3_), 3.38 ppm (3H, CH_3_-O), 3.64 ppm (4mH, CH_2_-CH_2_-O), 3.77 ppm (4nH, CH_2_-O), 4.2 ppm (4nH, CH_2_-O-C(O)). Size exclusion chromathography (SEC): (dimethylformamide (DMF, poly(ethylene glycol) standards): Mn = 8300 g mol^−1^, *Đ* = 1.09.

The mPEG-b-PTMC copolymer was synthesized under similar conditions. ^1^H NMR (CDCl_3_, 600 MHz): δ 2.05 ppm (2nH, CH_2_-CH_2_-O-C(O)), 3.38 ppm (3H, CH_3_-O), 3.64 ppm (4mH, CH_2_-CH_2_-O), 4.24 ppm (4nH, CH_2_-O-C(O)). SEC: (DMF, poly(ethylene glycol) standards): M_n_ = 9000 g mol^−1^, *Đ* = 1.05.

### 2.3. Characterization of Synthetic Copolymers

The ^1^H NMR spectra were recorded on a Bruker-Avance II 600 MHz spectrometer with CDCl_3_ as the solvent, and tetramethylsilane was applied as the internal standard. SEC measurements were carried out in DMF with the addition of 5 mM LiBr at a flow rate of 1 mL·min^−1^ at 45 °C. The chromatography system equipped with a multiangle light scattering detector (MALS, DAWN HELEOS, Wyatt Technology, λ = 658 nm), a refractive index detector (Dn-2010 RI, WGE Dr Bures), and a column system (PSS gel GRAM guard and PSS GRAM 100 Å, 1000 Å and 3000 Å, respectively). Poly(ethylene glycol) standards (M_n_ values ranging from 1010 to 29,600 g·mol^−1^, Polymer Laboratories) were used for calibration.

### 2.4. Preparation and Characterization of Micelles

The micelles were prepared using the solvent evaporation method, as previously described [29]. Briefly, the respective copolymer was dissolved in dry CH_2_Cl_2_. Then, the solvent was evaporated to obtain a copolymer thin film at the bottom of the vial. After that, the film was dispersed in phosphate buffered saline (PBS) buffer, the mixture was vigorously stirred for 30 min and sonicated for 30–60 min in an ice-cold bath. After the solution clarified, the micelle suspension was filtered through a syringe filter (0.2 μm). The prepared micelle (mPEG-b-PKPC^mic^ or mPEG-b-PTMC^mic^) solution was lyophilized and stored in the glovebox freezer. The hydrodynamic diameter and size distribution of mPEG-b-PKPC^mic^ and mPEG-b-PTMC^mic^ were determined by dynamic light scattering (DLS) measurements using a Zetasizer Nano ZS90 (Malvern Instruments). The critical micelle concentration (CMC) was determined using pyrene as a fluorescence probe. Fluorescence measurements were recorded on the Hitachi F-2500 Spectrometer (Tokyo, Japan), with the emission wavelength at 391 nm, in the range from 300 to 360 nm. The intensity ratio of pyrene probe at 336 over 333 nm (I_336_/I_333_) was plotted against the logarithm of the appropriate copolymer concentration to determine the CMC. The characteristics of the morphology of micelles were obtained using Cryogenic Transmission Electron Microscopy (cryo-TEM). Images were recorded using a Tecnai F20 X TWIN microscope (FEI Company, Hillsboro, OR, USA), operating at an acceleration voltage of 200 kV. Images were acquired with the Gatan Rio 16 CMOS 4k camera (Gatan Inc., Pleasanton, CA, USA), and processed with Gatan Microscopy Suite (GMS) software (Gatan Inc., Pleasanton, CA, USA). Vitrification of the aqueous solutions of specimens was carried out with holey carbon film grids (Quantifoil R 2/2; Quantifoil Micro Tools GmbH, Großlöbichau, Germany). Before use, the grids were activated for 15 s in oxygen plasma, using a Femto plasma cleaner (Diener Electronic, Ebhausen, Germany). Samples were prepared by applying 3 μL of the suspension to the grid and freezing it in liquid ethane, using a Vitrobot Mark IV (Thermo Fisher Scientific, Waltham, MA, USA). The vitrified specimens were inserted into a cryo-TEM-holder Gatan 626 (Gatan Inc., Pleasanton, CA, USA) and analyzed in the TEM at −178 °C.

### 2.5. Preparation and Characterization of Supramolecular Hydrogels

SMHs were prepared based on the host–guest interaction between the PEG chains of mPEG-b-PKPC or mPEG-b-PTMC micelles and α-CD. Typically, the lyophilized micelles were resuspended (15 mg·mL^−1^) in PBS at pH 7.4, as a hydrogel precursor. Then, the same volume of 12% solution of α-CD in PBS was added to the micelle solution. The mixture was vortexed for 1 min followed by sonication for 1 min. The resultant mixture was allowed to stand still overnight for solidification. Firstly, gelation was visually observed by a simple vial inversion method. When the mixture did not flow, gel formation was assumed. Thus, SMHs obtained (mPEG-b-PKPC^SMH^ or mPEG-b-PTMC^SMH^) were used for characterization studies without any further modification. The transmittance of the α-CD and mPEG-b-PKPC^mic^ solution, measured at predetermined time intervals at 650 nm, immediately after mixing, was studied using a UV-Vis spectrophotometer JASCO V-570 (Tokyo, Japan). To confirm the formation of supramolecular hydrogels, the samples of mPEG_5k_, α-CD, mPEG-b-PKPC, or mPEG-b-PTMC, the lyophilized mPEG-b-PKPC^mic^, or mPEG-b-PTMC^mic^, the physical mixture of α-CD with the respective micelles, and freeze-dried SMHs were studied using the X-ray diffraction method (XRD). The XRD measurements were performed using the D8 Advance diffractometer (Bruker, Karlsruhe, Germany) with a Cu-Kα cathode (λ = 1.54 Å), using Bragg-Brentano geometry. The scan rate was 1.2°/min with scanning step 0.01° in a range of 5° to 55° 2Θ. The formation of SMHs was further confirmed by Differential Scanning Calorimeter (DSC). DSC studies were done using a TA DSC Q2000 apparatus (TA Instruments, New Castle, DE, USA) calibrated with high-purity indium. The thermal characteristics of samples were carried out at a temperature ranging from −20 °C to 180 °C, with a heating rate of 20 °C/min. The experiments were performed under an inert nitrogen atmosphere with a nitrogen flow rate of 50 mL/min. Rheological tests were performed using a Brookfield RST-CPS rheometer, in a cone-plate measuring system (75 mm cone diameter, 1° angle) at a temperature of 37 °C. The shear rate was swept logarithmically from ~0.04 s^−1^ to 1000 s^−1^ in 310 s (0.845 decades/min) and then back to ~0.04 s^−1^, at the same rate. To study the stability and acid-triggered degradation of SMHs at various pH, the 5 cm^3^ cylinder-shaped mPEG-b-PKPC^SMH^ or mPEG-b-PTMC^SMH^ were incubated at 37 °C in 50 mL of PBS buffer, with pH 7.4, 6.4, or 5.5 for 24 and 48 h. After the hydrogel samples had been aged, the corresponding buffer was poured out and the sample was carefully transferred to the rheometer. The morphology of SMH was observed using the high-resolution scanning electron microscope (SEM) Quanta 250 FEG (FEI Company, Fremont, CA, USA). The samples were prepared without coating and the analysis was performed under a low vacuum (80 Pa). The accelerating voltage used was equal to 5 kV. The freshly prepared SMHs were frozen in liquid nitrogen and freeze-dried. Then, the lyophilized SMHs were split, and the interior morphology studied. The NMR titration was used to investigate the possibility of the formation of host–guest inclusion complexes of 8HQ-Glu with α-CD. The experiments were carried out in D_2_O, keeping the concentration of the α-CD constant at 10 mM, while varying the 8HQ-Glu concentration from 1 to 20 mM. The samples were incubated for 24 h at rt with gentle shaking and the NMR spectra were measured.

### 2.6. Preparation of DOX/8HQ-Glu-Loaded Supramolecular Hydrogels

DOX-loaded micelles were prepared with the same procedure as the preparation of the blank micelles, using 10 mg of the corresponding copolymer and 1 mg of DOX. Afterward, the unloaded drug was eliminated by centrifugation. The supernatant was recovered and filtered through a syringe filter (0.2 μm), freeze-dried, and stored in a glovebox freezer. The drug loading efficiency (DLE) and drug loading content (DLC) were calculated using the following equations:

$$\left(\mathrm{DLE}\right)=\frac{\mathrm{weight}\;\mathrm{of}\;\mathrm{drug}\;\mathrm{in}\;\mathrm{micelles}}{\mathrm{weight}\;\mathrm{of}\;\mathrm{drug}\;\mathrm{in}\;\mathrm{feed}}\times 100\%$$

$$\left(\mathrm{DLC}\right)=\frac{\mathrm{weight}\;\mathrm{of}\;\mathrm{drug}\;\mathrm{in}\;\mathrm{micelles}}{\mathrm{weight}\;\mathrm{of}\;\mathrm{drug}\;\mathrm{loaded}\;\mathrm{micelles}\;}\times 100\%$$

Then, in order to obtain dual drug-loaded SMHs, 10 mL of 10% α-CD solution containing 2 mg of glycoconjugate was added to 10 mL of DOX-loaded micelles. The mixture was vortexed, sonicated, and incubated overnight at 4 °C still for solidification. In vitro drug release studies from SMHs were carried out at 37 °C in PBS at pH 7.4, 6.4, and 5.5 using a dialysis method. Typically, a 5 cm^3^ cylinder-shaped dual drug-loaded supramolecular hydrogel was transferred into a Slide-A-Lyzer Mini Dialysis Device (MWCO 3500, Thermo Fisher Scientific). Each device was placed in a falcon tube with 50 mL of corresponding PBS buffer. Then, the device was placed in an incubator at 37 °C with gentle shaking. At the predetermined time intervals, the PBS buffer was taken out from the falcon tube and replaced with an equal volume of fresh buffer. The quantitative assessment of DOX and 8HQ-Glu released from the SMHs was determined by UV-Vis spectroscopy at 481 nm and 260 nm, respectively.

### 2.7. MTT Assay

The cytotoxicity of blank and drug-loaded SMH were evaluated using the MTT assay (Merck, Darmstadt, Germany). Briefly, human breast adenocarcinoma (MCF-7) or Normal Human Dermal Fibroblasts-Neonatal (NHDF-Neo) cells were seeded into 96-well plates at a concentration of 5 × 10^3^ per well. The cell cultures were incubated for 24 h at 37 °C in a humidified atmosphere of 5% CO_2_. Afterward, the culture medium was removed, replaced with tested formulations, in a medium with varying concentrations, and incubated further for 24, 48, or 72 h, respectively. After that, the medium was removed and the MTT solution was added (0.5 mg·mL^−1^ in PBS). After three hours of incubation, the MTT solution was taken off and the precipitated formazan was dissolved in dimethylsulfoxide. The absorbance was measured at 570 nm using a plate reader (Epoch, BioTek, Winooski, VT, USA). The experiments were performed in at least three independent repetitions, with four technical repeats for each tested concentration. The IC_50_ values were calculated using CalcuSyn software (ver. 2.0, Biosoft, Cambridge, UK). The IC_50_ parameter was defined as the compound concentration that was necessary to reduce the proliferation of cells to 50% of the untreated control. The results are presented as average values with standard deviations. Furthermore, the synergistic effect of DOX and 8HQ-Glu was evaluated by the Chou–Talalay combination index (CI) method calculated using the following equation [40]:

$$\mathrm{CI}=\frac{{\left(D\right)}_{1}}{{\left(\mathrm{Dx}\right)}_{1}}+\frac{{\left(D\right)}_{2}}{{\left(\mathrm{Dx}\right)}_{2}}$$

where (D)_1_ and (D)_2_ are the respective combination doses of drug 1 and drug 2 that yield an effect of 50% proliferation inhibition (IC_50_), with (Dx)_1_ and (Dx)_2_ being the corresponding single doses for drug 1 and drug 2 that also inhibit proliferation by 50%. Values of CI < 1 represent synergism; CI = 1 represents an additive interaction (i.e., no interaction); and CI > 1 represents antagonism.

## 3. Results and Discussion

### 3.1. Copolymer Synthesis and Characterization

**Figure 1:**
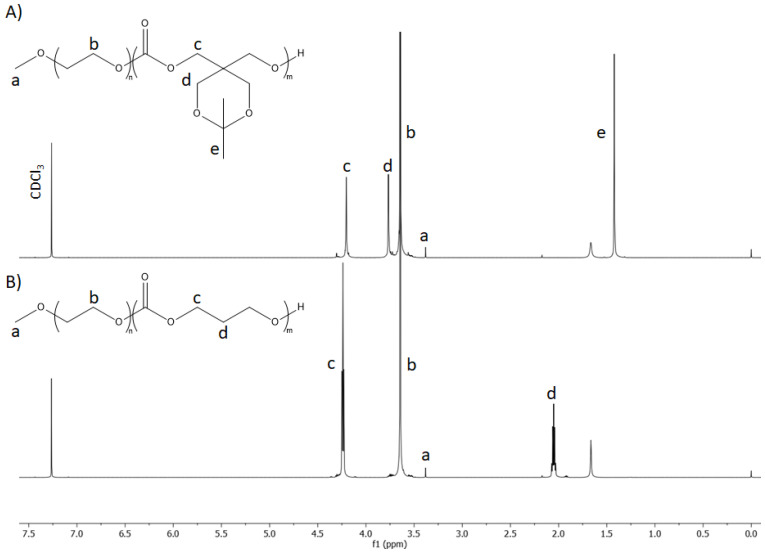
^1^H NMR spectrum (CDCl_3_, 600 MHz) of: (**A**) mPEG-b-PKPC and (**B**) mPEG-b-PTMC.

To take advantage of the acidic tumor microenvironment, we developed a pH-responsive supramolecular hydrogel for codelivery of 8HQ-Glu and DOX for enhanced tumor treatment. To construct a designed supramolecular hydrogel system, a pH-sensitive copolymer was first prepared by the introduction of ketal groups into the hydrophobic part of the amphiphilic copolymer. Afterward, such obtained amphiphilic copolymers were used to form micelles. The ketal groups underwent acid-triggered hydrolysis to form two hydroxyl groups, increasing the hydrophilicity of the hydrophobic micelle core, causing swelling of the nanoparticles and the release of the loaded drug. The PEGylated ketal-protected aliphatic polycarbonate diblock copolymer (mPEG-b-PKPC) was synthesized via controlled anionic ring-opening polymerization of ketal-functionalized six-membered cyclic carbonate monomer (9,9-dimethyl-2,4,8,10-tetraoxaspiro[5.5]undecan-3-one). The monomethoxy-poly(ethylene glycol), with a molar mass of 5000 g mol^−1^, was used as an initiator, and DBU/TU as a catalytic system. The theoretical molar mass of the hydrophobic PKPC block was specified to be 4000 g mol^−1^ to keep the hydrophilic mPEG block weight fraction around 60%, since copolymers with hydrophilic block weight fractions around 0.45–0.70 are reported as micelles forming [41]. The ^1^H NMR spectrum of mPEG-b-PKPC is shown in Figure 1A. All of the main characteristic signals assigned to methoxy-poly(ethylene glycol) (δ = 3.38 and 3.64 ppm) and aliphatic polycarbonate (δ = 1.42, 3.77, 4.2 ppm) are observed.

The molar mass of mPEG-b-PKPC was calculated based on the integral ratio between NMR resonances of the mPEG methylene protons and the methylene protons of aliphatic polycarbonate. Using these integral ratios with the molar mass of mPEG = 5000 g mol^−1^, the molar mass of mPEG-b-PKPC was determined to be 8600 g mol^−1^. The value correlates with the theoretically estimated 9000 g mol^−1^. SEC analysis (Figure S2) showed that the synthesized copolymer had a molar mass of 8300 g mol^−1^ and a narrow dispersity as *Đ* = 1.09. The mPEG-*b*-PTMC diblock copolymer (Figure 1B), with a similar molar mass, was synthesized under similar conditions and used as a pH-insensitive control in all further studies. The results of the ^1^H NMR and SEC studies for the obtained copolymers are summarized in Table 1.

### 3.2. Preparation and Characterization of Micelles

**Figure 2:**
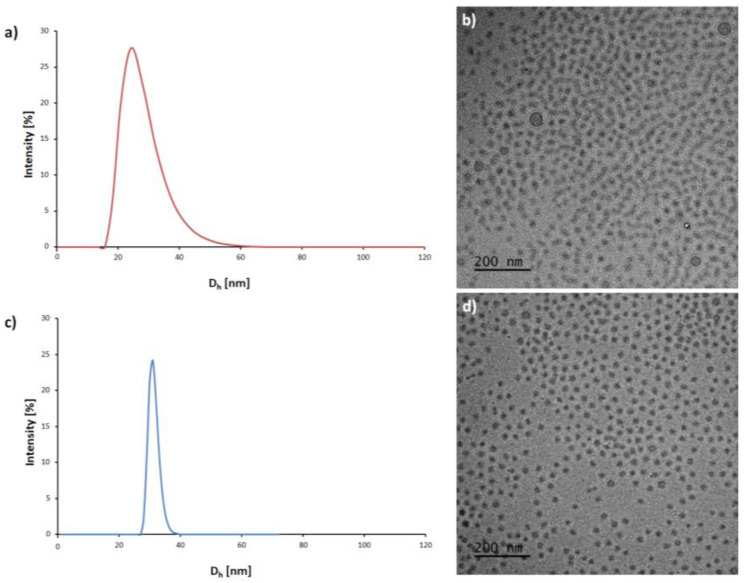
The size distributions and cryo-TEM images of mPEG-b-PKPC^mic^ (**a**,**b**) and mPEG-b-PTMC^mic^ (**c**,**d**).

The resulting amphiphilic diblock copolymers spontaneously formed core-shell type micelles by self-organization, when exposed to an aqueous environment. The critical micelle concentration was determined as a parameter to reflect the stability of micelles in aqueous media. The critical micelle concentration was measured using pyrene as a fluorescence probe, from the plot of the excitation intensity ratio as a function of the corresponding amphiphilic copolymer concentration. The CMC values of mPEG-b-PKPC^mic^ and mPEG-b-PTMC^mic^ were determined to be 0.038 mg·mL^−1^ and 0.012 mg·mL^−1^, respectively (Figure S3). The size and morphology of the micelles were characterized by DLS measurement and cryo-TEM studies, respectively. DLS studies showed that mPEG-b-PKPC formed micelles with an average size of ~27 nm (Figure 2a). The spherical shape of mPEG-b-PKPC^mic^ was confirmed by cryo-TEM with an average size of ~20 nm (Figure 2b). The average size of mPEG-b-PKPC^mic^, determined by cryo-TEM studies, was slightly less than the values measured by DLS. The reason for this is that DLS reflects the hydrodynamic diameter in the solution, while the size observed by cryo-TEM indicates that of dried micelles [42]. The mPEG-b-PTMC^mic^ displayed a hydrodynamic diameter ~31 nm and spherical shape (Figure 2c,d), therefore allowing it to be used as a control for further research.

### 3.3. Preparation and Characterization of Supramolecular Hydrogels

**Figure 3:**
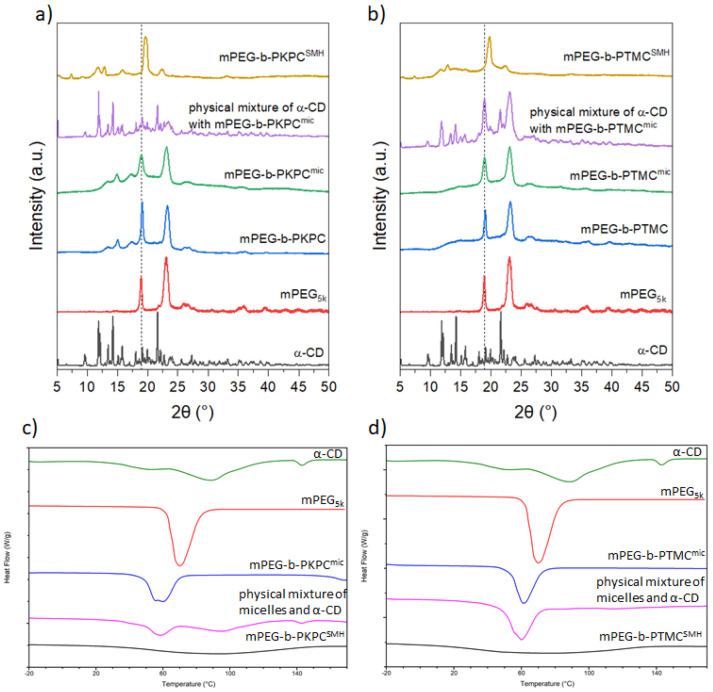
X-ray diffractograms for (**a**) mPEG-b-PKPC and (**b**) mPEG-b-PTMC series; DSC curves for (**c**) mPEG-b-PKPC and (**d**) mPEG-b-PTMC series.

The linear PEG can penetrate the inner cavity of α-CD via host–guest interactions to form crystalline inclusion complexes. These inclusion complexes tend to aggregate into channel-type microcrystals, acting as physical crosslink points leading to the formation of a supramolecular hydrogel. In the present supramolecular system, pH-responsive micelles with a hydrophobic core and outer shell containing hydrophilic PEG chains were used as first-level crosslink points. Afterward, the poly(pseudo)rotaxanes, formed by PEG chains on micelle surfaces and α-CD, aggregates into chan-nel-type microcrystals, and act as the second level physical cross-link points, prompting the formation of a supra-molecular hydrogel network. Scheme 1 shows the schematic illustration of formation, both drugs loading, and environment triggered release from mPEG-b-PKPC^SMH^. The effect of the α-CD and PEG concentrations on the formation of SMHs has been widely studied in the literature [43,44,45]. According to recent studies, the most beneficial systems for controlled drug delivery based on micelles/α-CD were obtained with 10–15 mg·mL^−1^ micelles concentrations and concentrations of α-CD not exceeding 9% [7]. Herein, hydrogel samples were obtained by mixing equal amounts of micelle solutions (concentration of 15 mg·mL^−1^) and α-CD with a concentration varying from 14%, 12%, and 10%, respectively (14.5% is the maximum concentration of α-CD in water at rt [46]). Thus, the concentration of micelles in the final supramolecular hydrogels was constant at 7.5 mg·mL^−1^, and the concentration of CD varied to 7%, 6%, and 5%, respectively. Gel formation was observed in all cases, in accordance with the literature and due to appropriate rheological properties (*vide infra*), SMHs with micelle concentrations of 7.5 mg·mL^−1^ and α-CD 6% was used for further studies [47]. At interval times, the appearances of turbidity changes of the resulted inclusion complexes at different intermediate states are presented in Figure S4, displaying the clear contrast between the starting mixture and the final crosslinked state. The transmittance of the α-CD and mPEG-b-PKPC^mic^ transparent mixture decreased from 95% to 3% in 3 min (Figure S4), which revealed the formation and aggregation of poly(pseudo)rotaxanes. To confirm inclusion complex formation between micelle derived PEG chains and α-CD, X-ray diffraction measurements were conducted for α-CD, mPEG_5k_, freeze-dried micelles, physical mixture of micelles with α-CD, and the resultant freeze-dried SMH (Figure 3a). In the XRD pattern of mPEG-b-PKPC and mPEG-b-PKPC^mic^, the two characteristic peaks at 19.1° and 23.2° from the mPEG crystalline phase were ascribed. The XRD pattern for α-CD was characteristic of multiple diffraction peaks corresponding to its crystalline form. The physical mixture of mPEG-b-PKPC^mic^ and α-CD, the XRD spectra show the overlay of the pattern of α-CD and micelles, strongly suggesting a lack of host–guest interactions. The XRD pattern for freeze-dried mPEG-b-PKPC^SMH^ revealed a disappearance of both peaks for the PEG crystalline phase and showed a characteristic for PEG/α-CD inclusion complex peak at 2θ = 19.7°. This unique feature indicates that the channel-type microcrystalline inclusion complexes were formed by threading α-CD onto PEG chains by host–guest interactions [43]. The same results were also observed for control mPEG-b-PTMC^SMH^ samples (Figure 3b).

Furthermore, the DSC studies were also performed to examine the formation of the PEG/α-CD inclusion complex. The DSC curves of α-CD, micelles, their physical mixture, and lyophilized SMH are shown in Figure 3c,d. The physical mixture of mPEG-b-PKPC^mic^ with α-CD showed peaks corresponding to the overlay of the copolymer and α-CD curves, indicating no inclusion complex formation. The endothermic peaks noticed in the α-CD and micelles disappeared in the lyophilized mPEG-b-PKPC^SMH^. Essentially, the absence of the endotherm peak corresponding to the PEG chains melting, suggests poly(pseudo)rotaxane formation, because the PEG chains in the lyophilized supramolecular hydrogel are enclosed in the α-CD cavity and are protected against the melting behavior [43,44].

**Figure 4:**
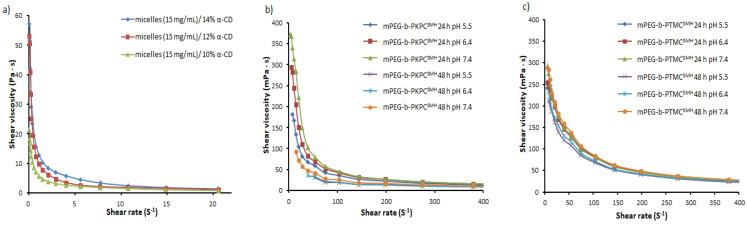
(**a**) Effect of different α-CD concentrations on the mPEG-b-PKCP^SMH^ shear viscosity (37 °C). Effect of incubation of (**b**) mPEG-b-PKCP^SMH^ or (**c**) mPEG-b-PTMC^SMH^ at different pH (7.4, 6.4, and 5.5) at 37 °C for 24 and 48 h on shear viscosity.

The shear-thinning studies were carried out to determine the rheological behavior of supramolecular hydrogels. As shown in Figure 4a the viscosity of the mPEG-b-PKPC^SMH^ dropped rapidly with the increase of shear rates, indicating shear-thinning behavior. The thixotropic property is ascribed to the dynamic nature of physical host–guest interactions [48]. The supramolecular hydrogel collapses by shear forces, due to the disintegration of inclusion complexes between α-CD and PEG chains, which significantly reduces the crosslink point numbers in the supramolecular matrix. The thixotropic behavior might be beneficial for injectable drug delivery systems. Supramolecular hydrogels might be injected into a specific place under shear forces, and, after that, turned in situ in SMHs when withdrawing the shear forces, without losing its designed controlled drug release properties. Additionally, the viscosity of the mPEG-b-PKPC^SMH^ significantly increases with the increase of α-CD concentration, thus the viscosity of the resulting supramolecular system can be easily tailored with the α-CD concentration.

Figure 4b,c shows the rheological behavior of mPEG-b-PKPC^SMH^ and control mPEG-b-PTMC^SMH^ as a function of shear rate during the incubation of SMH in physiological conditions at various pH. It is observed that the initial viscosity of SMHs drastically decreased after incubation. The decrease in viscosity after incubation is caused by the erosion of the supramolecular hydrogel matrix, resulting from the dissolution of α-CD, which induces decomplexation and subsequent SMH disintegration [44]. It is worth noting that after incubation, both supramolecular hydrogels kept their initial shape, with traces of external erosion. The slight pH-dependent effect of viscosity reduction during incubation has been observed for mPEG-b-PKPC^SMH^ compared to control mPEG-b-PTMC^SMH^. It was attributed to acid-triggered hydrolysis of ketal groups causing the micelles to swell and further collapse, decreasing the number of crosslinks, thus breaking down of SMH matrix. Surprisingly, a pH-dependent matrix erosion effect during incubation was not observed in mPEG-b-PTMC^SMH^, although the α-CD/PEG host–guest complexation constant depends on the pH [49]. At lowered pH levels, the host–guest complexation constant decreased, resulting in the decomplexation of the PEG/α-CD during incubation, leading to gel breakdown faster at pH 5.5, than at 7.4. The above results suggest that the matrix erosion effect caused by the dissolution of α-CD is much bigger than the pH effect.

**Figure 5:**
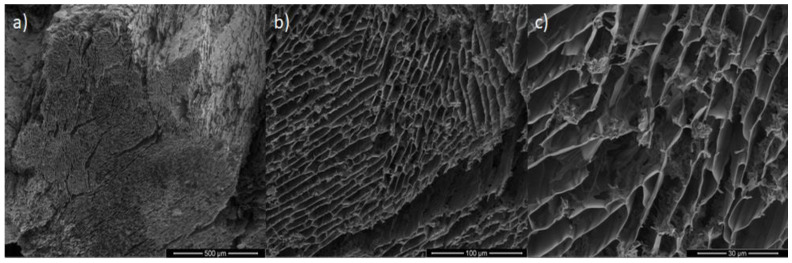
SEM images (various magnification, (**a**–**c**)) of the freeze-dried mPEG-b-PKPC^SMH^.

The morphology of mPEG-b-PKPC^SMH^ was observed by SEM measurement. As shown in Figure 5a–c the freeze-dried SMH reveals a highly homogeneous porous structure due to the high water content.

### 3.4. In Vitro Drug Loading and Release

**Figure 6:**
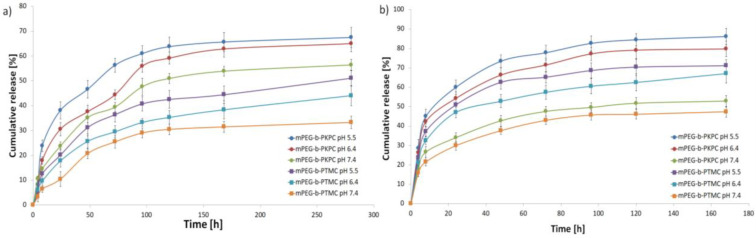
In vitro release profiles for (**a**) DOX and (**b**) 8HQ-Glu from mPEG-b-PKPC^SMH^ and mPEG-b-PTMC^SMH^ in PBS buffers at 37 °C. Data are presented as the mean ± SD (*n* = 3).

In vitro drug release studies were performed to evaluate the ability of mPEG-b-PKPC^SMH^ to efficiently codeliver both hydrophilic and hydrophobic drugs. Two different kinds of chemotherapeutic agents, 8HQ-Glu and DOX, were physically loaded into a supramolecular hydrogel matrix. Hydrophobic DOX was encapsulated into micelles, and hydrophilic 8HQ-Glu was dispersed directly in a hydrogel matrix. At first, the NMR titration was used to investigate the possibility of the formation of host–guest inclusion complexes of 8HQ-Glu with α-CD. By analyzing the chemical shifts of α-CD signals (mainly H3 and H5 at δ = 4.03 and 3.95 ppm, respectively), the possibility of complex formation was determined because the change in the chemical shift is evidence of host–guest inclusion complex formation [50]. For the 8HQ-Glu/α-CD system, no shift of α-CD signals was observed as shown in Figure 5, indicating no inclusion complex formation. As a result, 8HQ-Glu could be efficiently loaded into the SMH matrix. The hydrophobic DOX was successfully loaded into micelles, where the drug loading efficiency and drug loading content were determined as 49.1 ± 2.9% and 5.5 ± 0.4%, respectively. In vitro drug release profiles of mPEG-b-PKPC^SMH^ were investigated at 37 °C in three different PBS buffers at pH 7.4, 6.4, and 5.5, respectively. The results revealed that DOX was released from mPEG-b-PKPC^SMH^ faster under acidic conditions than under physiological conditions as shown in Figure 6a. DOX was released from mPEG-b-PKPC^SMH^ at levels of 38% and 66% after 24 and 168 h at pH 5.5, respectively, whereas 30% and 63% were released at pH 6.4 under the same conditions. At pH 7.4, about 54% of DOX release was observed after 168 h. The pH-dependent release behavior was ascribed to the acid accelerated ketal group hydrolysis to two hydroxyl groups, leading to an increase in hydrophilicity of the hydrophobic micelle core, causing the mPEG-b-PKPC^mic^ to swell and initiating encapsulated DOX release [29]. The control mPEG-b-PTMC^SMH^ revealed a significantly slower release of DOX in all studied pH conditions. The slightly increased DOX release rates in acidic conditions for non-pH-responsive SMH are most likely due to DOX solubility significantly increasing with lowered pH [51]. Figure 6b demonstrates the release of hydrophilic 8HQ-Glu physically entrapped in hydrogel matrix from mPEG-b-PKPC^SMH^ compared to control mPEG-b-PTMC^SMH^. The hydrophilic 8HQ-Glu release was faster compared to DOX release profiles and more favorable in acidic conditions. Therefore, 73% and 86% of 8HQ-Glu were released from mPEG-b-PKPC^SMH^ in 48 and 168 h, respectively, at pH 5.5 and 66% and 80% after the same time at pH 6.4. Furthermore, the 43% and 52% of 8HQ-Glu were released within 48 and 168 h at pH 7.4. In acidic conditions, the 8HQ-Glu release rate was still accelerated for mPEG-b-PTMC^SHM^. At pH 5.5, approximately 62% and 71% of 8HQ-Glu were released within 48 and 168 h, respectively. For comparison, about 38% and 47% of 8HQ-Glu were released after incubation within 48 and 168 h at physiological pH. The relatively faster release of both drugs in the acidic environment is due to the looser structure of the SMH, caused by the decrease in the PEG/α-CD host–guest complexation constant. On the other hand, significantly accelerated drug release, especially DOX for mPEG-b-PKPC^SMH^, is additionally caused by a second effect i.e., the pH-triggered decomposition of micelles, which reduces the crosslink point numbers in the supramolecular hydrogel matrix. Notably, the DOX was released at a slower rate compared to 8HQ-Glu. This phenomenon is ascribed to SMH matrix dissociation, leading to the release of 8HQ-Glu and DOX-loaded micelles, which subsequently release the loaded drug, prolonging the release of DOX. The results demonstrate that both hydrophilic and hydrophobic drugs can be loaded into the mPEG-b-PKPC^SMH^ and released in a controlled manner, within the slightly acidic tumor microenvironment.

### 3.5. In Vitro Cytotoxicity

To evaluate the synergistic effect of the co-loaded supramolecular hydrogel, in vitro cytotoxicity was studied against MCF-7 (human breast adenocarcinoma cell line) and NHDF-Neo (Normal Human Dermal Fibroblasts-Neonatal) cells using MTT assay. Cells were incubated with the free drugs or drugs-loaded mPEG-b-PKPC^SMH^ for 24, 48, and 72 h in a concentration range oscillating within their IC_50_ activity. Firstly, the blank mPEG-b-PKPC^SMH^ was tested and no cytotoxicity effect on MCF-7 and NHDF-Neo cells was found.

**Figure 7:**
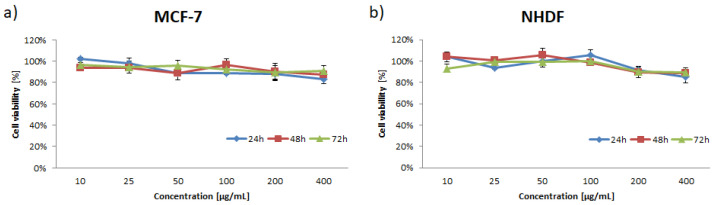
Cytotoxicity of the blank mPEG-b-PKPC^SMH^ determined by MTT assay with MCF-7 and NHDF cells. Data are presented as the mean ± SD (*n* = 3).

**Figure 8:**
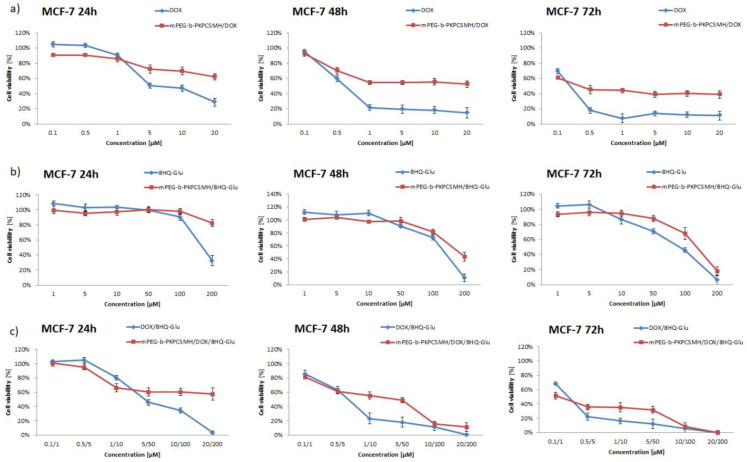
Cell viability of MCF-7 cells treated with: (**a**) DOX and mPEG-b-PKPC^SMH^/DOX; (**b**) 8HQ-Glu and mPEG-b-PKPC^SMH^/8HQ-Glu; and (**c**) DOX/8HQ-Glu (1:10) and mPEG-b-PKPC^SMH^/DOX/8HQ-Glu. Data are presented as the mean ± SD (*n* = 3).

**Figure 9:**
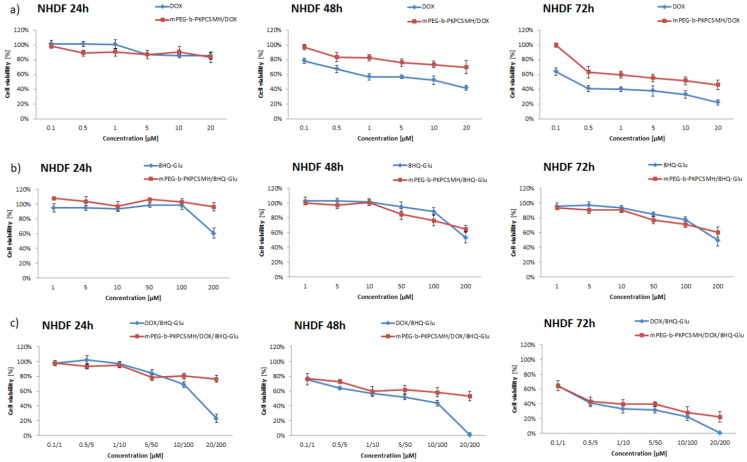
Cell viability of NHDF cells treated with: (**a**) DOX and mPEG-b-PKPC^SMH^/DOX; (**b**) 8HQ-Glu and mPEG-b-PKPC^SMH^/8HQ-Glu; and (**c**) DOX/8HQ-Glu (1:10) and mPEG-b-PKPC^SMH^/DOX/8HQ-Glu. Data are presented as the mean ± SD (*n* = 3).

As shown in Figure 7 the viability of MCF-7 or NHDF-Neo cells during the incubation with blank supramolecular hydrogel remained in the range of 88–102% or 85–106%, respectively. Therefore, the blank mPEG-b-PKPC^SMH^ is biocompatible with tested cell lines and can be safely used for research as a drug delivery system. The effect of the pH-responsive supramolecular hydrogel matrix was assessed by comparing the cytotoxicity of free drugs and drugs-loaded mPEG-b-PKPC^SMH^. As displayed in Figure 8 and Figure 9, free drugs and drugs-loaded mPEG-b-PKPC^SMH^ show dose-dependent cell growth inhibition of MCF-7 and NHDF-Neo cells. The combination of DOX and 8HQ-Glu resulted in the enhanced cytotoxicity effect. It was found that IC_50_ of free DOX or free 8HQ-Glu was 8.73 or 167.9 µM, against MCF-7 cells after 24 h of incubation, respectively. While the IC_50_ decreased to 4.95 µM for DOX and 49.51 µM for 8HQ-Glu when using the DOX/8HQ-Glu (1:10) mixture, the dose of used drugs could be reduced by 43.3% and 70.5%, respectively. This result suggested that codelivery of DOX and 8HQ-Glu may be a favorable strategy to reduce the dose of anti-cancer drugs, thereby reducing side effects, and improving the treatment effect.

It was observed that after 24 h of incubation, the proliferation inhibition rates of drugs-loaded mPEG-b-PKPC^SMH^ were lower compared to free drugs. As the incubation time was prolonged, the cytotoxicity effect of drugs-loaded mPEG-b-PKPC^SMH^ was increased and was close to the cytotoxicity effect corresponding to free drugs. This effect was more noticeable with mPEG-b-PKPC^SMH^ loaded with hydrophilic glycoconjugate or the DOX/8HQ-Glu drug mixture. This is because the dissociation of mPEG-b-PKPC^SMH^ matrix leads to the release of the free 8HQ-Glu and DOX-loaded micelles. The 8HQ-Glu has the potential to easily penetrate the cancer cells by active transport through GLUT transporters [39]. Meanwhile, the pH-sensitive micelles might release encapsulated DOX in a slightly acidic tumor extracellular microenvironment, or DOX-loaded micelles enter the cancer cells via endocytosis, which prolongs the anti-cancer activity of DOX. As shown in Table 2, when compared with MCF-7 cancer cells incubated with the same drug formulations, healthy NHDF-Neo cells showed significantly higher IC_50_ values. The higher cytotoxicity effect in all tested formulations for MCF-7 cancer cells might be related to the Warburg effect. The slightly acidic environment near the cancer cells might prompt faster release of both drugs from pH-responsive mPEG-b-PKPC^SMH^. Moreover, overexpression of GLUT transporters near cancer cells should facilitate the penetration of 8HQ-Glu into cancer cells, improving anti-cancer activity.

Furthermore, the CI (combination index) was used to evaluate the synergistic action of DOX and 8HQ-Glu on MCF-7 and NHDF cell inhibition. The synergistic effect is generally determined as follows: CI < 1 is a synergistic action; CI > 1 is an antagonistic action and CI = 1 is an additive action [52]. As shown in Table 3, the synergistic interaction of DOX and 8HQ-Glu showed dose and cell type dependent properties. The CI was >1 in the earlier incubation stages for free DOX/8HQ-Glu mixture or mPEG-b-PKPCS^MH^/DOX-8HQ-Glu demonstrated an antagonistic effect against both cancer and healthy cell lines, likely caused by a low dose of drugs. The CI values decreased with increasing incubation time, showing that DOX/8HQ-Glu has better synergism efficiency at higher drugs dose levels. This effect was observed faster against MCF-7 cells incubated with mPEG-b-PKPC^SMH^/DOX-8HQ-Glu, which might suggest faster drugs release within the cancer cell microenvironment. Moreover, CI was <1 for MCF-7 cells incubated with free DOX/8HQ-Glu mixture for 24 and 48 h, while after 72 h at high dose effect, the antagonistic effect was observed. This suggests that appropriate combinations of DOX and 8HQ-Glu could be established, which would improve the antitumor efficacy. Moreover, the dual-drug loaded mPEG-b-PKPC^SMH^ display a sustainable drug release profile, suggesting that SMHs are beneficial carriers for an efficient DOX and 8HQ-Glu codelivery system.

## 4. Conclusions

In this study, pH-responsive supramolecular hydrogel mPEG-b-PKPC^SMH^ as a delivery carrier for DOX and 8HQ-Glu was fabricated by the two-level self-assembly of PEGylated ketal protected polycarbonate, followed by poly(pseudo)rotaxane formation by α-CD and PEG chains. The amphiphilic mPEG-b-PKPC copolymer self-assembly in aqueous media to form micelles with an average diameter of ~27 nm was determined by DLS and cryo-TEM. The poly(pseudo)rotaxane formation was confirmed by both XRD and DSC studies. The drugs-loaded mPEG-b-PKPC^SMH^ displayed accelerated drugs release profiles in an acidic environment, due to acid-triggered hydrolysis of ketal bonds and reducing α-CD/PEG host–guest complexation constant. It was found that the combination of DOX and 8HQ-Glu suppressed the proliferation of cancer cells more effectively than the two single drugs alone, which is attributed to a synergistic effect. In vitro studies demonstrated that the mPEG-b-PKCP^SMH^ is non-toxic toward cancer MCF-7 cells, and more importantly against healthy NHDF-Neo cells. Moreover, a sustained inhibition efficacy towards cancer cells was determined using the mPEG-b-PKPC^SMH^/DOX-8HQGlu, compared with the free drugs mixture. The strategy of codelivery of DOX and 8HQ-Glycoconjugates via pH-responsive supramolecular hydrogel matrix may be beneficial to increase the selectivity of anti-cancer therapy, and reduce the dose of drugs, thereby reducing side effects, and improving the treatment effect.

## Figures and Tables

**Table 1:** Characteristics of mPEG-b-PKPC and mPEG-b-PTMC copolymers.

Block Copolymer	M_n,NMR_^a^ [g mol^−1^]	M_n,SEC_^b^ [g mol^−1^]	*Đ*	*f*_PEG_ [%]
mPEG-*b*-PKPC	8600	8300	1.09	60
mPEG-*b*-PTMC	9500	9400	1.05	53

^a^ The mPEG block had M_n_ = 5000 and *Đ* = 1.01 (determined by SEC). ^b^ Determined by SEC with DMF as solvent and poly(ethylene glycol) standards.

**Table 2:** Screening of cytotoxicity of tested formulations against MCF-7 and NHDF-Neo cells for 24, 48, and 72 h incubation time.

Compound	Activity IC_50_ [µM] ^a^					
	MCF-7					
	24 h		48 h		72 h	
	DOX	8HQ-Glu	DOX	8HQ-Glu	DOX	8HQ-Glu
**DOX**	8.73	-	0.81	-	0.15	-
**mPEG-b-PKPC^SMH^/DOX**	>20	-	>20	-	0.46	-
**8HQ-Glu**	-	167.9	-	127.6	-	55.32
**mPEG-b-PKPC^SMH^/8HQ-Glu**	-	>200	-	188.33	-	125.73
**DOX/8HQ-Glu**	4.95	49.51	0.61	6.14	0.23	2.3
**mPEG-b-PKPC^SMH^/DOX/8HQ-Glu**	>20	>200	1.38	13.81	0.15	1.54
**Compound**	**Activity IC_50_ [µM] ^a^**					
	**NHDF**					
	**24 h**		**48 h**		**72 h**	
	**DOX**	**8HQ-Glu**	**DOX**	**8HQ-Glu**	**DOX**	**8HQ-Glu**
**DOX**	>20	-	9.8	-	0.39	-
**mPEG-b-PKPC^SMH^/DOX**	>20	-	>20	-	11.77	-
**8HQ-Glu**	-	>200	-	>200	-	>200
**mPEG-b-PKPC^SMH^/8HQ-Glu**	-	>200	-	>200	-	>200
**DOX/8HQ-Glu**	12.22	122.27	4.39	43.99	0.28	2.82
**mPEG-b-PKPC^SMH^/DOX/8HQ-Glu**	>20	>200	>20	>200	0.36	3.68

^a^ Cytotoxicity was evaluated using the MTT assay.

**Table 3:** Combination index analysis. CI < 1—synergism, CI > 1—antagonism, and CI = 1 additive effect.

	Combination Index					
	MCF-7			NHDF		
	24 h	48 h	72 h	24 h	48 h	72 h
**DOX/8HQ-Glu**	0.86	0.8	1.58	>1	<1	<1
**mPEG-b-PKPC^SMH^/DOX/8HQ-Glu**	>1	<1	0.36	>1	>1	<1

## Data Availability

Not applicable.

## Supplementary Materials

The following supporting information can be downloaded at: https://www.mdpi.com/article/10.3390/pharmaceutics14112490/s1, Figure S1: Structure of glycoconjugate 6-(4-(8-quinolinyloxymethyl)-1H-1,2,3-triazol-1-yl)-6-deoxy-D-glucopyranose (8HQ-Glu); Figure S2: Size exclusion chromatograms of mPEG5k, mPEG-b-PKPC and mPEG-b-PTMC (in DMF, calibrated against PEG standards); Figure S3: Self-assembled critical micelle concentration of (a) mPEG-b-PKPC and (b) mPEG-PKPC-b-PTMC; Figure S4: Time-dependent solution transmittance plot of mPEG-b-PKPC^mic^ (15 mg·mL^−1^) and α-CD (10%) mixture; Figure S5: ^1^H NMR (600 MHz, D_2_O) spectra of α-CD and 8HQ-Glu and their mixture at different molar ratios.
