# Supplementary material for: Co-Delivery of 8-Hydroxyquinoline Glycoconjugates and Doxorubicin by Supramolecular Hydrogel Based on α-Cyclodextrin and pH-Responsive Micelles for Enhanced Tumor Treatment
Source: Pharmaceutics. 2022 Nov 17;14(11):2490. doi: 10.3390/pharmaceutics14112490 (PMC9697330; doi:10.3390/pharmaceutics14112490)
Supplement: Supplementary file 1 [file pharmaceutics-14-02490-s001.zip › pharmaceutics-2005292-supplementary.pdf]

Supporting Information

Co-delivery of 8-Hydroxyquinoline Glycoconjugates and Doxorubicin by Supramolecular Hydrogel from α-Cyclodextrin and pH-responsive Micelles for Enhanced Tumor Treatment

Adrian Domiński[a]*, Tomasz Konieczny[a], Marcin Godzierz[a], Marta Musioł[a], Henryk Janeczek[a], Aleksander Foryś[a], Monika Domińska[b,c], Gabriela Pastuch-Gawołek[b,c], Tomasz Piotrowski[d], Piotr Kurcok[a],*

[a] Centre of Polymer and Carbon Materials, Polish Academy of Sciences, 34, M. Curie-Skłodowskiej St, 41-819 Zabrze, Poland

[b] Department of Organic Chemistry, Bioorganic Chemistry and Biotechnology, Faculty of Chemistry, Silesian University of Technology, Krzywoustego 4, 44-100 Gliwice, Poland

[c] Biotechnology Centre, Silesian University of Technology, B. Krzywoustego 8, 44-100 Gliwice, Poland

[d] Department of Chemical Organic Technology and Petrochemistry, Faculty of Chemistry, Silesian University of Technology, Bolesława Krzywoustego 4, 44-1100 Gliwice, Poland

*Correspondence: piotr.kurcok@cmpw-pan.edu.pl, adominski@cmpw-pan.edu.pl

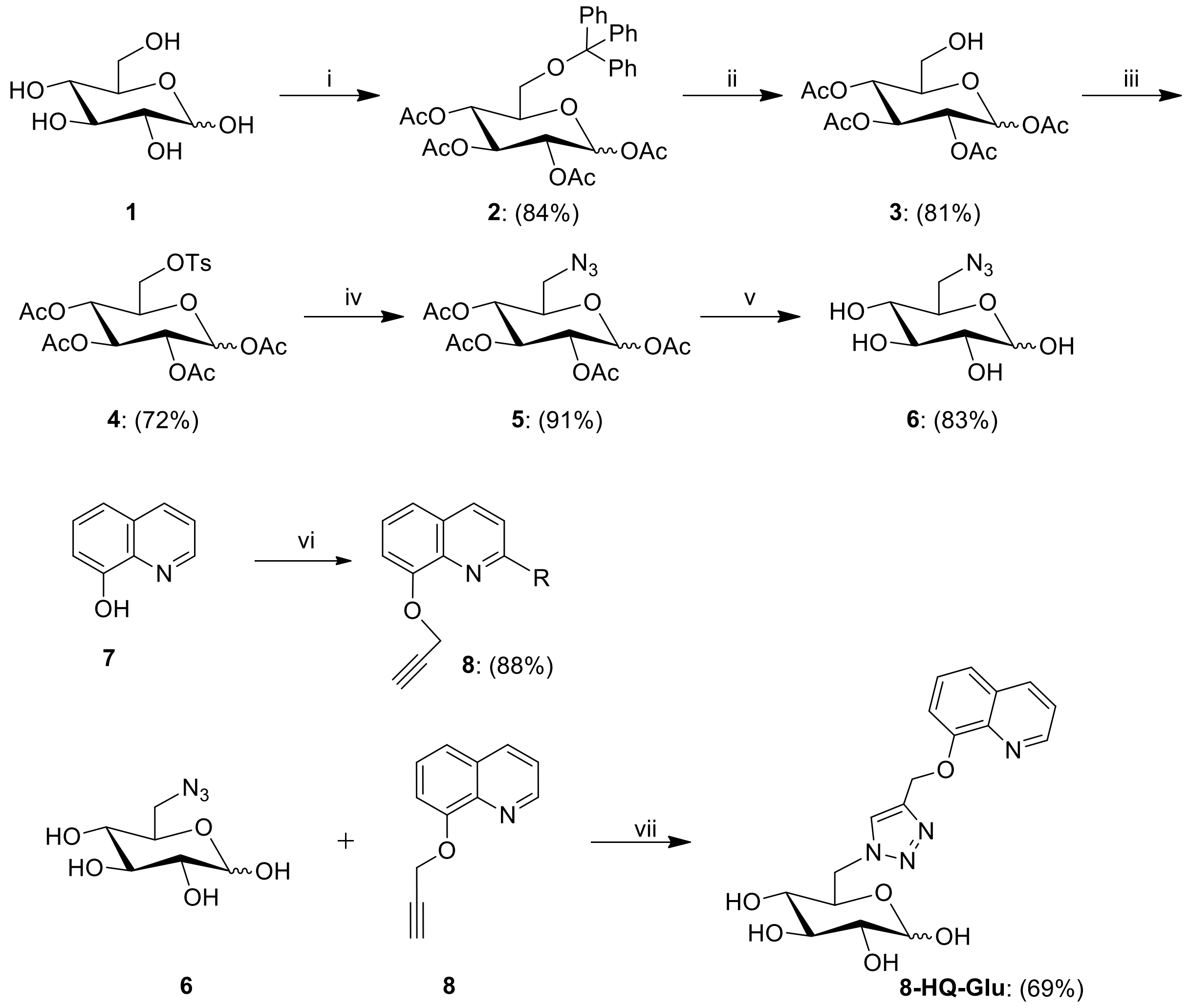

Fig. S1. Synthesis of 8HQ-Glu (6-(4-(8-quinolinyloxymethyl)-1H-1,2,3-triazol-1-yl)-6-deoxy-D-glucopyranose). Reagents and Conditions: (i) 1. trityl chloride, DMAP, pyridine, r.t., 24 h; 2. CH_3COCl, pyridine, r.t., 1h; (ii) CH_3COOH, 33% HBr/AcOH, 0 °C-r.t., 1 h; (iii) p-TsCl, DMAP, pyridine, r.t., 24 h; (iv) NaN_3, DMF, 80 °C, 2 h; (v) 1. MeONa, MeOH, r.t., 0.5 h; 2. Amberlyst-15; (vi) propargyl bromide, K_2CO_3, acetone, r.t., 24 h; (vii) $CuSO_4{\cdot}5H_2O$, NaAsc, i-PrOH/THF/H_2O (1:1:1, v:v:v), r.t., 24 h.

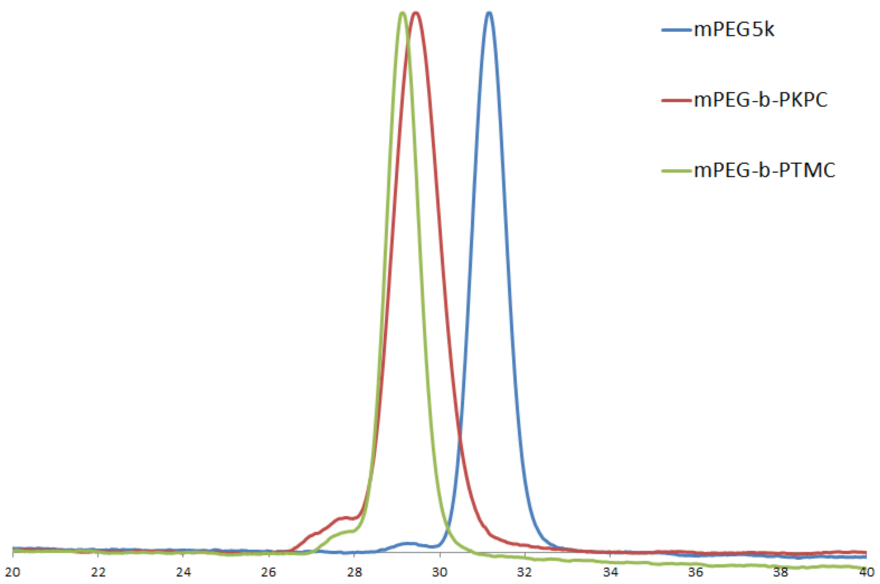

Fig. S2. Size exclusion chromatograms of mPEG5k, mPEG-*b*-PKPC and mPEG-b-PTMC (in DMF, calibrated against PEG standards).

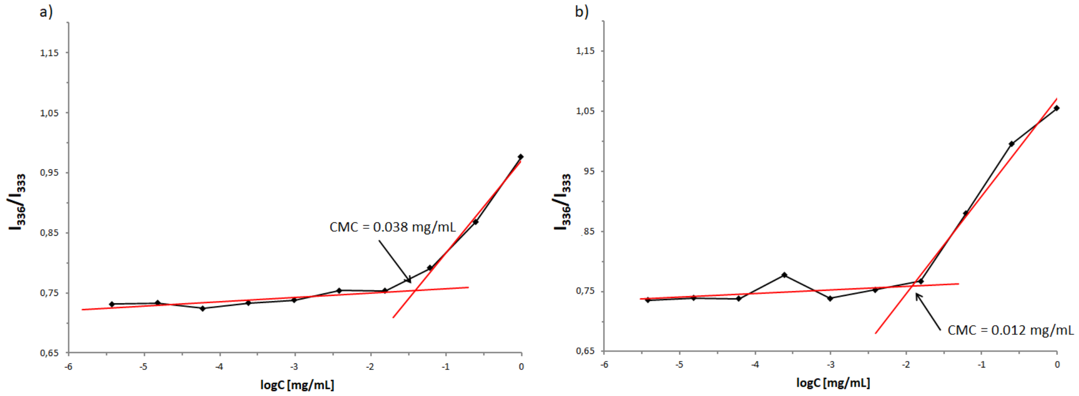

Fig. S3. Self-assembled critical micelle concentration of a) mPEG-*b*-PKPC and b) mPEG-PKPC-*b*-PTMC.

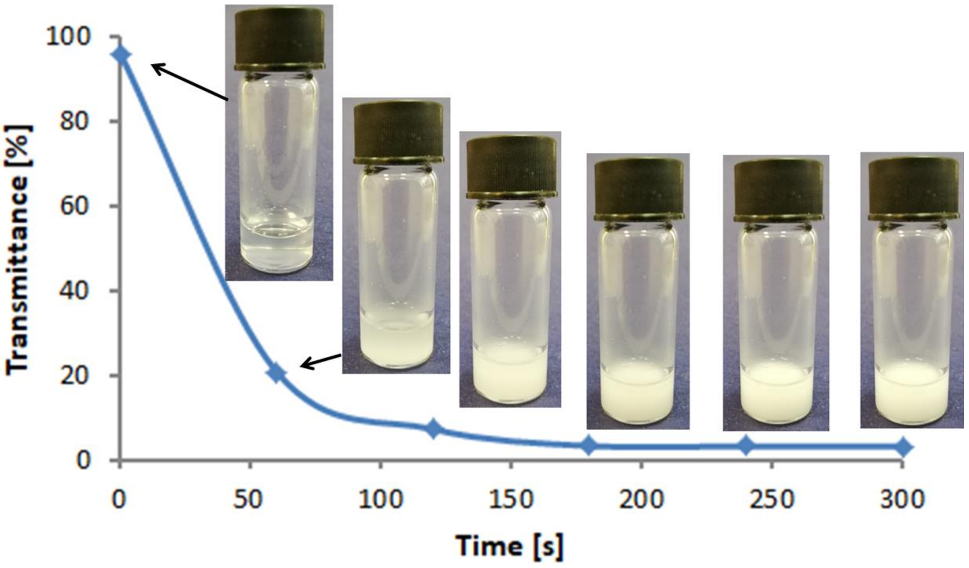

Fig. S4. Time-dependent solution transmittance plot of mPEG-b-PKPC^{mic} (15 mg mL^{-1}) and α-CD (10%) mixture.

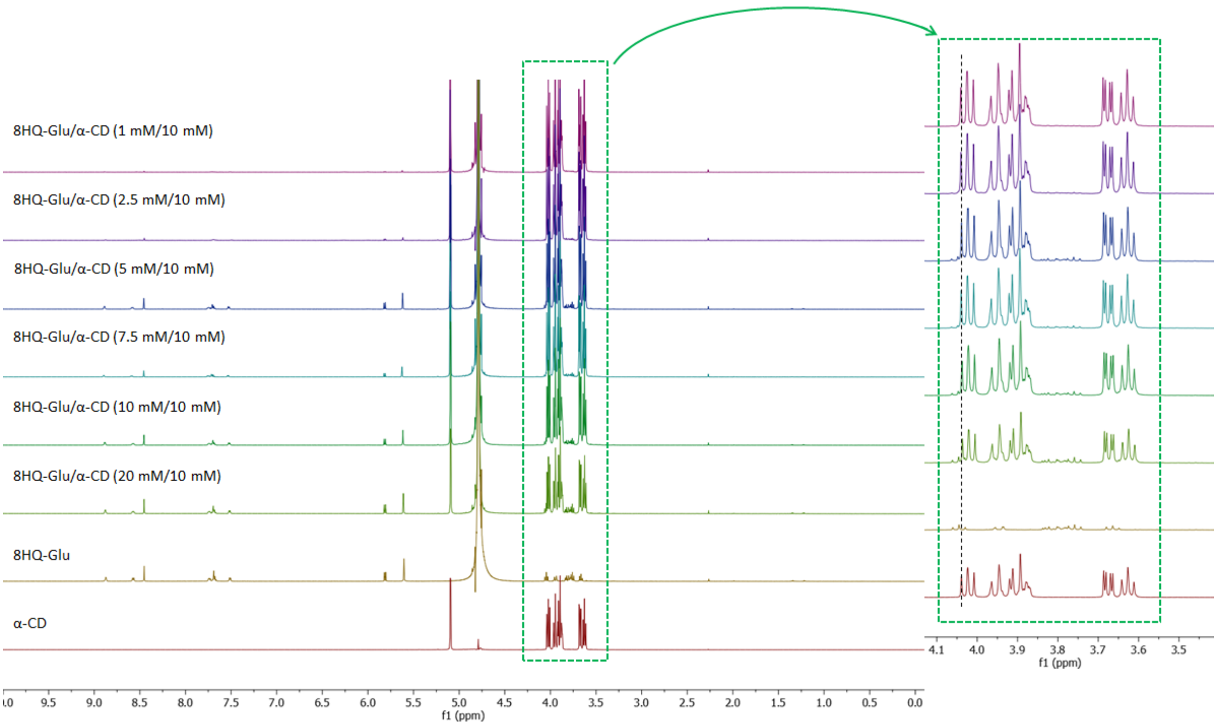

Fig. S5. ^{1}H NMR (600 MHz, D_2O) spectra of α-CD and 8HQ-Glu and their mixture at different molar ratios.
